# Complete mitochondrial genome sequence and phylogenetic analysis of *Lycodes polaris* (Sabine, 1824)

**DOI:** 10.1080/23802359.2019.1624650

**Published:** 2019-07-11

**Authors:** Hai Li, Yuan Li, Ji Feng, Puqing Song, Nan Zhang, Longshan Lin

**Affiliations:** Laboratory of Marine Biology and Ecology, Third Institute of Oceanography, Ministry of Natural Resouces, Xiamen, P. R. China

**Keywords:** *Lycodes polaris*, mitochondrial genome, Arctic fish, Zoarcidae

## Abstract

*Lycodes polaris* is one of the most widely distributed and abundant eelpout species on the Arctic continental shelves with full mitogenome information unavailable. In this paper, complete mitochondrial genome of *L. Polaris* was determined with 16,595 bp in length, containing 13 protein-coding genes, 2 ribosomal RNA genes, 22 transfer RNA genes, and 2 non-coding regions (origin of light strand replication and control region). All the protein-coding genes choose ATG as start codon in addition to that COI using GTG. Most genes use TAA or TAG as the stop codon while three others ended with incomplete stop codons. Phylogenetic relationship of *L. polaris* was also reconstructed. Our result will provide important basis for further studies of *L. polaris*.

The Canadian eelpout, or also known as polar eelpout, *Lycodes polaris* (Sabine, 1824) belongs to the family Zoarcidae (Mecklenburg et al. 2013; Norcross et al. 2013). It occurs nearly circumpolar along the Arctic coasts of Asia and North America, which starts from Barents Sea, eastward through Siberian and North American Arctic, and then westward to Greenland and southward to Gulf of St. Lawrence and northern Bering Sea, respectively (Mecklenburg et al. 2016). As one of the most widely distributed and abundant eelpout species on the Arctic continental shelves (Rand and Logerwell, 2011; Mecklenburg and Steinke 2015), however, its full mitochondrial genome information is unavailable. In this paper, we report the complete mitochondrial genome of this species, additional phylogenetic analysis is also conducted in the study.

The voucher specimen was captured from Chukchi Sea continental shelf (168.671°W, 73.697°N) during the 8^th^ Chinese National Arctic Research Expedition in 2017, and deposited in the Third Institute of Oceanography, Ministry of Natural Resources.

Long PCR and primer walking approaches were both employed to amplify the complete sequences (Genbank accession No. MK910376) with 29 pairs of primers. The mitogenome of *L. polaris* is 16,595 bp in length consisting of 2 ribosomal RNA (12S and 16S) genes, 22 transfer RNA genes (tRNA), 13 protein-coding genes (PCGs), and 2 non-coding regions (control region and light-strand replication origin). With the exception of ND6 and eight tRNAs (-Gln, -Ala, -Asn, -Cys, -Tyr, -Ser, -Glu, -Pro), rest mitochondrial genes are all encoded on the heavy strand. The base composition of four nucleotides is 25.7% (A), 18.2% (G), 25.1% (T), 30.6% (C), respectively. All PCGs but COI (GTG) choose ATG as the start codon. Three PCGs ended with incomplete stop codons (COII, ND4, Cyt *b*), and the other genes ended either with TAG (ND1, ND2, ND3, ND5) or TAA (COI, ATPase8, ATPase6, CO III, ND4L, ND6).

To further verify the phylogenetic position of *L. polaris*, a neighbor-joining (NJ) tree was constructed on the basis of the complete mitogenome sequences of both *L. polaris* and other five Arctic fish species using MEGA 6.06 (Tamura et al. 2013). The result suggests that all fish species were apparently distinct with each other with high bootstrap values (Figure 1). Meanwhile, *L. Polaris* was more closed with two other Zoarcidae species, and then clustered with two Cottidae species and a Anarhichadidae species, respectively.

**Figure 1:**
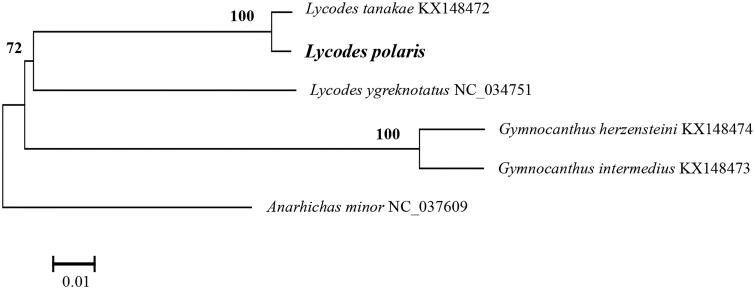
Phylogenetic relationship of *L. polaris*. The cited mitogenome sequenced are downloaded from GenBank and the phylogenic tree is constructed by the neighbor-joining method with 100 bootstrap replicates. Bootstrap values of >50% are shown above the node.

The complete mitochondrial genome analysis and reconstructed phylogenetic relationship of *L. polaris* report in our paper is believed to provide important information regarded to molecular phylogenetics, population genetics, and conservation biology.
